# The Role of LGR4 (GPR48) in Normal and Cancer Processes

**DOI:** 10.3390/ijms22094690

**Published:** 2021-04-29

**Authors:** Alejandro Ordaz-Ramos, Victor Hugo Rosales-Gallegos, Jorge Melendez-Zajgla, Vilma Maldonado, Karla Vazquez-Santillan

**Affiliations:** 1 Posgrado en Ciencias Biológicas, Universidad Nacional Autónoma de México, Mexico City 04510, Mexico; alex-mdna@ciencias.unam.mx (A.O.-R.); vh.rosales@ciencias.unam.mx (V.H.R.-G.); 2 Epigenectics Laboratory, Instituto Nacional de Medicina Genómica, Mexico City 14610, Mexico; vmaldonado@inmegen.gob.mx; 3 Functional Genomics Laboratory, Instituto Nacional de Medicina Genómica, Mexico City 14610, Mexico; jmelendez@inmegen.gob.mx

**Keywords:** LGR4, GPR48, cancer, CSCs

## Abstract

Leucine-rich repeats containing G protein-coupled receptor 4 (LGR4) is a receptor that belongs to the superfamily of G protein-coupled receptors that can be activated by R-spondins (RSPOs), Norrin, circLGR4, and the ligand of the receptor activator of nuclear factor kappa-B (RANKL) ligands to regulate signaling pathways in normal and pathological processes. LGR4 is widely expressed in different tissues where it has multiple functions such as tissue development and maintenance. LGR4 mainly acts through the Wnt/β-catenin pathway to regulate proliferation, survival, and differentiation. In cancer, LGR4 participates in tumor progression, invasion, and metastasis. Furthermore, recent evidence reveals that LGR4 is essential for the regulation of the cancer stem cell population by controlling self-renewal and regulating stem cell properties. This review summarizes the function of LGR4 and its ligands in normal and malignant processes.

## 1. Introduction

Cancer is one of the major burdens of disease worldwide. Cancer development involves genetic and epigenetic alterations that allow cells to escape from the mechanisms that control proliferation and survival. Many of these alterations correspond to signaling pathways that regulate multiple cellular processes such as cell growth, cell death, fate, and motility [1]. G protein-coupled receptors (GPCRs) are one of the largest superfamilies of cell-surface receptors involved in membrane-initiated signaling processes. GPCRs share various structural characteristics including an extracellular N-terminal domain, seven transmembrane domains connected with extra- and intra- cellular loops, and an intracellular C-terminal domain. Many types of GPCRs have been described in humans, which have key roles in a variety of physiological and pathological processes [2]. In cancer, GPCRs participate in a plethora of processes such as proliferation, migration, apoptosis, and tumorigenesis [3].

Leucine-rich repeats containing G protein-coupled receptors (LGRs) are a group of transmembrane receptors that belong to the GPCRs superfamily, characterized by a large extracellular domain that recognizes ligands and regulates numerous cellular processes. LGRs are classified into three groups according to their function and structure. Group A receptors include LGR1, which recognizes follicle-stimulating hormone (FSH), LGR2, which recognizes luteinizing hormone (LH), and LGR3, which recognizes thyroid-stimulating hormone (TSH). Group B includes LGR4, LGR5, and LGR6 receptors, which play crucial roles in developmental processes and are involved in several types of cancer. Finally, Group C includes LGR7 (RXFP1 receptor) and LGR8 (RXFP2 receptor) recognizing relaxin and insulin-like peptide 3 (INSL3) [4,5].

LGR4 has a large extracellular Leucine-rich domain that is capable of interacting with its ligands. LGR4 is commonly activated by RSPOs, Norrin, Receptor activator of NF-kappa B ligand (RANKL), and circLGR4 ligands, and its activation results in the signaling of the Wnt/β-catenin and G protein-associated pathways [6,7,8,9,10,11]. Accumulating evidence indicates that LGR4 expression is upregulated in cancer tissues and participates in the regulation of various tumorigenic processes. In this review, we describe the characteristics of LGR4 signaling pathways and their implication in normal and malignant cellular processes.

## 2. LGR4 Characterization

LGR4 is a transmembrane receptor member of the GPCRs superfamily and belongs to group B of the LGR family [4]. LGR4 was first characterized in 1998 as homologous to the well-known members of the LH/FSH/TSH family of receptors (Group A) [12]. LGR4 is encoded by a highly conserved 106,827 pb gene, located on human chromosome 11 (11p14.1). The genomic organization of the LGR4 gene involves 18 exons that are subjected to alternative splicing resulting in two isoforms, one of them encoding a protein of 951aa [12,13,14]. 

**Figure 1:**
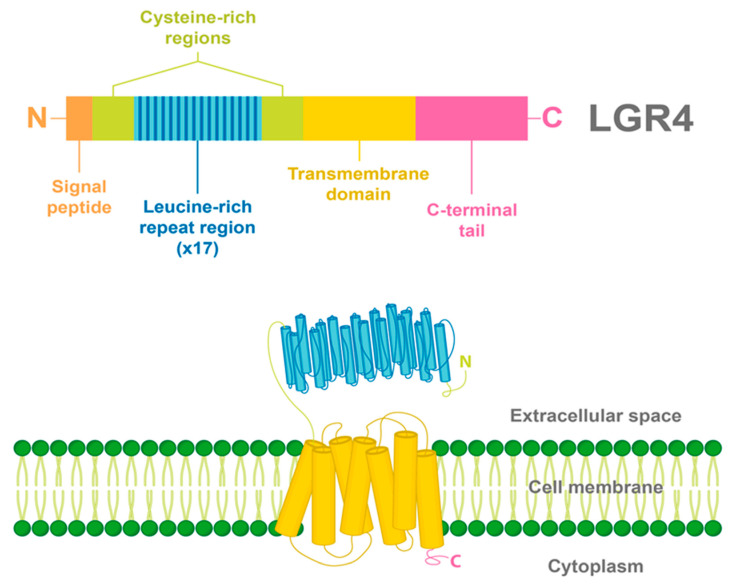
Structure and domains of LGR4. LGR4 is a transmembrane receptor with a long N-terminal extracellular domain constituted by 17 leucine-rich repeats, flanked by cysteine-rich regions. In addition, a seven-transmembrane helix domain and a C-terminal intracellular domain are found in LGR4. The signal peptide of LGR4 is found in the N-terminal region.

Similar to all LGRs, LGR4 has a large N-terminal extracellular domain that enables the binding of specific ligands. This extracellular domain is constituted by 17 leucine-rich repeats (LRR) flanked by N-/C- cysteine-rich regions. A common seven-transmembrane helix domain characteristic of all GPCRs is found in LGR4, having three extracellular and three intracellular loops, and a C-terminal intracellular domain [12,14] (Figure 1).

LGR4 is widely expressed in several tissues including the mammary gland, bone, prostate, skin, pancreas, ovary, heart, kidney, testis, brain, thymus, among others [12,14,15]. The interaction of this receptor with its ligands modulates signaling pathways associated with physiological and developmental processes. 

## 3. Ligands and Canonical Signaling Pathways Regulated by LGR4

### 3.1. R-Spondins (RSPOs)

LGR4 was considered an orphan receptor until 2011, when Carmon et al., Glynca et al., and Ruffner et al. performed co-immunoprecipitation, co-immunofluorescence, and binding assays to show that RSPOs bind LGR4 and modulate the Wnt signaling pathway [6,7,11].

**Figure 2:**
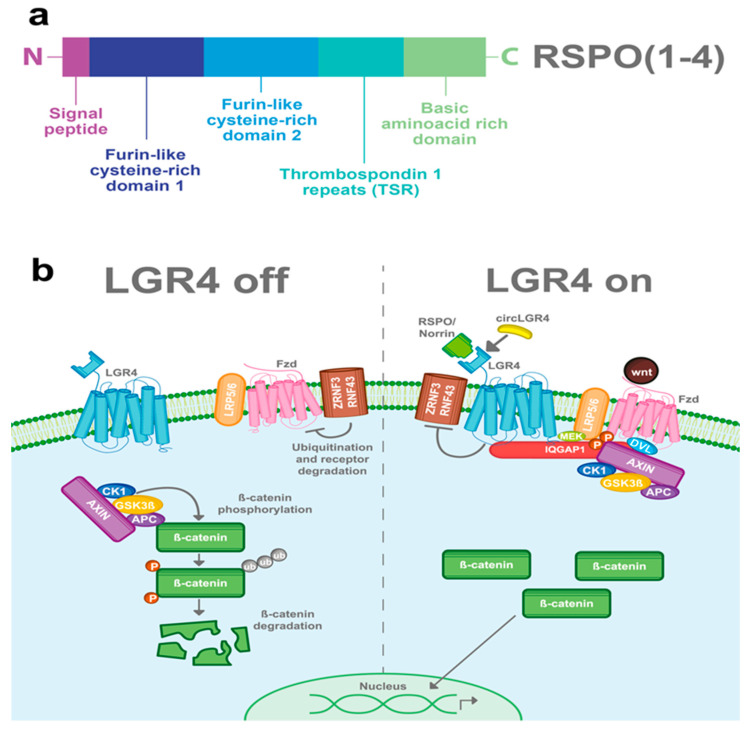
LGR4-induced Wnt/β-catenin signaling pathway. (**a**) RSPOs are a family of secreted proteins that can activate LGR4-induced Wnt/β-catenin signaling. Structurally, all the RSPOs have a signal peptide in the N-terminal domain, two furin-like cysteine-rich domains, a thrombospondin 1 repeat domain (TSR), and a basic amino acid-rich domain, which varies in size according to the RSPO member (**b**) In the absence of RSPO, ZNF3/RNF43 ubiquitinates the frizzled (Fzd)/LRP5-6 receptor complex for degradation. Wnt signal is blocked and the β-catenin destruction complex (formed by CK1, GSK3β, APC, and AXIN) is activated. GSK3β and CK1 phosphorylate β-catenin, inducing its ubiquitination and consequent proteasomal degradation. When LGR4 is activated by RSPOs, Norrin, or circLGR4 ligands, it stabilizes the frizzled/Lrp5-6 complex in the membrane, avoiding its degradation by inhibiting the activity of ZNRF3 and RNF43 proteins. Furthermore, LGR4 recruits IQGAP1 with an increasing affinity for DVL and recruits MEK, which phosphorylates LRP5/6, leading to the recruitment and inhibition of the β-catenin destruction complex into the Fzd/Lrp5-6 complex receptor.

R-spondins (roof plate-specific spondin) are a family of four secreted proteins (RSPOs 1-4) that were characterized for the first time in mice. These proteins share about 40–60% of amino acid identity between them and also have homologous structures [16]. Structurally, RSPOs have (1) a signal peptide at the N-terminus for secretion; (2) two furin-like cysteine-rich domains that are necessary for ligand activity; (3) a thrombospondin 1 repeat domain (TSR); and (4) a basic amino acid-rich domain with different lengths at the C-terminus [16,17,18] (Figure 2a). RSPOs can activate three of the eight members of the LGR’s family (LGR4/5/6) and can induce a potent and sustained activation of the Wnt pathway, a well-known signaling cascade involved in several physiological and pathological processes including cancer [6,7,11,19].

The Wnt signaling pathway is mainly activated by the frizzled/LRP5-6 receptor complex. LGR4, through its extracellular domain, can interact with any of the four RSPOs and enhance the Wnt signaling pathway. Stimulation of LGR4 by RSPOs stabilizes the Frizzled/Lrp5-6 complex in the membrane, avoiding its degradation by inhibiting the activity of ZNRF3 and RNF43 proteins [20,21,22,23]. Furthermore, LGR4 recruits IQGAP1, a scaffold protein that induces the recruitment of the β-catenin destruction complex to the Fzd/LRP5-6 receptor complex [24,25]. The interaction of the Frizzled/LRP5-6 receptor complex with the Wnt ligands results in the cytoplasmic accumulation of β-catenin and its later nuclear translocation. In the nucleus, β-catenin, in a complex with the TCF transcription factor, acts as a transcription regulator of its target genes (Figure 2b) [26,27]. Interestingly, RSPOs can also enhance Wnt signaling independently of LGR receptors [16,17]. 

### 3.2. Norrin

In 2013, Deng et al. found a second ligand for LGR4 [8]. Norrin is a secreted protein that can promote Wnt signaling and regulate physiological processes. Structurally, Norrin has an N-terminal signal peptide and a cysteine-rich C-terminal domain that allows its homodimerization. Similar to RSPOs, Norrin can also interact with LGR4, stabilize the frizzled/LRP5-6 receptor complex and thus enhance the Wnt/β-catenin signal (Figure 2b). In addition, Norrin can also interact with frizzled receptors and intensify the Wnt pathway in an LGR4-independent way [8,28].

### 3.3. RANKL

**Figure 3:**
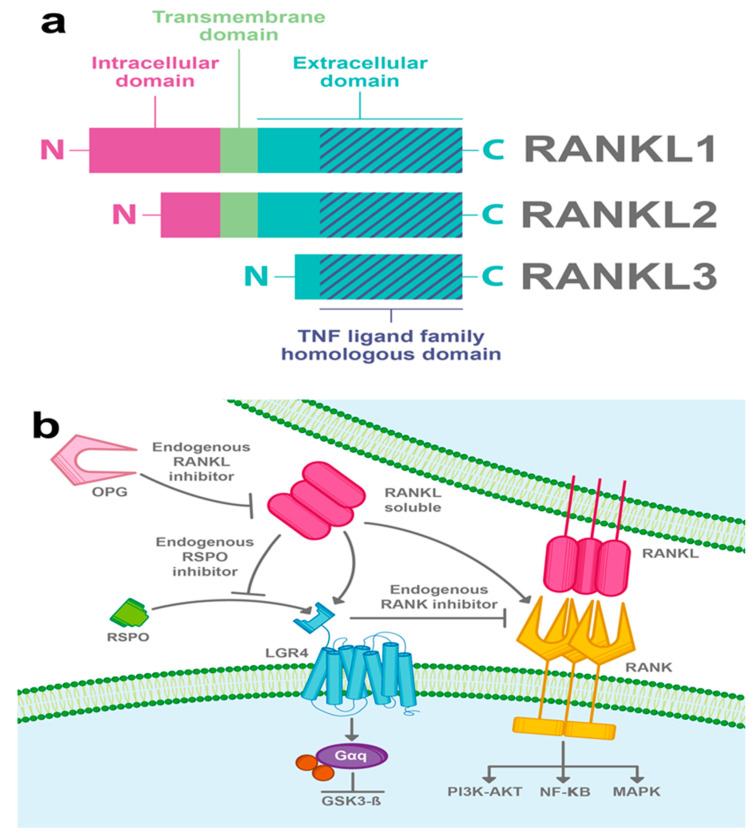
RANKL-induced LGR4 signaling pathway. (**a**) RANKL is another molecule that can promote LGR4 activity. Three isoforms of RANKL have been described; the full-length RANKL (RANKL1), a form lacking part of the intra-cytoplasmic domain (RANKL2), and a soluble form (RANKL3). All three isoforms have a TNF ligand family homologous domain in their extracellular part. (**b**) RANKL interacts with LGR4 and induces the Gαq protein pathway and also inhibits GSK3β. Furthermore, RANKL activates RANK and promotes NF-κB canonical RANK signaling among other pathways, for instance; PI3K-AKT and MAPK. LGR4 competes with RANK to bind RANKL and suppresses canonical RANK signaling. Besides, it has been suggested that RANKL can compete with RSPOs to bind LGR4 and in this way disrupt RSPO-induced Wnt/β-catenin signaling. Osteoprotegerin (OPG) acts as a RANKL endogenous inhibitor.

The ligand of the receptor activator of nuclear factor kappa-B (RANKL) is a homotrimeric type II membrane protein with no signal peptide, which belongs to the tumor necrosis factor (TNF) family of cytokines [29]. Three alternative spliced isoforms of RANKL have been detected; the full-length RANKL (RANKL1), a shorter form that lacks part of the cytoplasmic domain (RANKL2), and a soluble form with the N-terminal part deleted (RANKL3) (Figure 3a) [30]. RANKL was first characterized as a ligand of the RANK receptor, a well-known activator of the NF-κB signaling pathway, and both RANK and RANKL have been associated with bone remodeling by regulating osteoclast differentiation [29].

Since LGR4 has a typical GPCR structure, efforts have been made to search its G-protein-dependent function. In 2016, Luo et al. showed for the first time that RANKL binds to the extracellular domain of LGR4 and activates a Gαq protein that inhibits the GSK3β signaling pathway, thus suppressing the expression of the NFATC1, a key transcription factor for osteoclastogenesis (Figure 3b) [9]. 

It has been shown, in an osteoclast model, that LGR4 competes with RANK to bind RANKL and suppresses canonical RANK signaling, thus exerting an opposing effect on the RANK pathway. Interestingly, LGR4 is a downstream target of RANKL–RANK signaling, suggesting that LGR4 acts as a feedback loop controlling RANKL activities [9]. Furthermore, it has been suggested that RANKL can compete with RSPOs to bind LGR4 and, in this way, the interaction of RANKL/LGR4 can disrupt the Wnt/β-catenin signaling potentiated by RSPOs (Figure 3b) [9].

### 3.4. circLGR4

CircLGR4 is a circular RNA (circ-LGR4) that encodes a 19 amino acid peptide, which is secreted through the Golgi pathway. Recently, in a colorectal cancer model Zhi, et al. showed that the circLGR4 peptide interacts with the extracellular domain of LGR4 and enhances Wnt/β-catenin signaling (Figure 2b). Disruption of circLgr4 expression resulted in impaired colon cancer stem cell self-renewal, tumorigenesis, and invasion [10].

## 4. LGR4 Regulation through microRNAs

MicroRNAs are non-protein-coding, small (19–25 nucleotides) RNAs that execute their functions by regulating the expression of their target genes. MicroRNAs bind to the 3′ untranslated region of their target mRNAs, leading to translational repression or mRNA degradation [31]. Different microRNAs have been reported to regulate LGR4 expression in many experimental models. Mir-34 is a well-known family of microRNAs involved in normal and pathological processes. The mir-34 family has been reported to interact with LGR4 and regulate its expression in bone tissue, where miR-34c enhances osteoclast differentiation by targeting LGR4 [32]. Mir-34a and mir-34c can target LGR4 and enhance the inflammatory response of epidermal keratinocytes associated with venous ulcers by downregulation of the NF-κB signaling activity via the increment of p65 serine 468 phosphorylation [33]. LGR4 inhibition mediated by mir-34a can also modulate retinal pigment, epithelial proliferation, and migration [34].

LGR4 is also targeted by miR-137. Over-expression of this miRNA has been associated with an increased risk of fracture in patients with osteoporosis [35]. LGR4 expression is also regulated by the microRNA let-7b, leading to epithelial cell apoptosis in age-related cataracts [36].

It has been reported that microRNAs can regulate LGR4 expression during cancer development and progression. In prostate cancer, miR-218 prevents prostate cancer cell proliferation and invasion by inhibition of LGR4 [37]. MiR-137 also inhibits prostate cancer cell migration and epithelial-mesenchymal transition (EMT) by negatively regulating the epidermal growth factor receptor/extracellular signal-regulated kinase (EGFR/ERK) through LGR4 targeting [38]. In non-small-cell lung carcinoma, mir-449b targets LGR4 and thus prevents cell proliferation and invasion [39].

## 5. LGR4 in Normal Tissues

At the time LGR4 was characterized, this receptor was associated first with developmental processes. LGR4 is broadly expressed in some embryonic and adult tissues. In 2004, Mazerbourg et al. showed for the first time that LGR4 has an essential role in embryonic development. LGR4-null mice have a decreased intrauterine growth and increased embryonic lethality [40]. Based on these results, many studies have explored the role of LGR4 in organ function and development.

### 5.1. Male Reproductive Tract

LGR4 deficiency in male mice results in infertility. Mendive et al., in 2006, and Hoshii et al., in 2007, showed for the first time that LGR4-deficient male mice have problems in postnatal development of the reproductive tract. Knockout (KO) of LGR4 leads to tube elongation failure, reduction in cell proliferation, and morphological abnormalities in the testes and the epididymis in male mice. LGR4 deficiency also affects the transit of sperm and testicular fluid resulting in germinal epithelium atrophy, probably by the disruption of estrogen receptor (ESR1) expression [41,42,43]. In addition, loss of LGR4 affects the development of peritubular myoid cells and arrests germ cells at meiosis 1, thus reducing spermatogenesis mediated by the disruption of Wnt/β-catenin signaling [44].

LGR4 deficiency also decreases prostate stem cell proliferation and differentiation, decreasing the expression of Wnt, sonic hedgehog (Shh), and Notch1, consequently affecting prostate size, branching morphogenesis, and luminal epithelial cell enfolding [45].

### 5.2. Female Reproductive Tract

Accumulating evidence has shown that LGR4 is required for the postnatal development of uterine glands and to regulate the female reproductive tract in mice. LGR4 is expressed on the cell surface of the uterine epithelia and is constitutively expressed in the endometrium throughout the whole estrous cycle of mice [46]. Evidence has shown that LGR4 deletion reduces the fertility of female mice [47]. LGR4 deficiency leads to a lack of uterine progesterone receptor (PR) phosphorylation/activation and embryo implantation failure [48]. Besides, LGR4-deficient mice show altered epithelial differentiation characterized by a reduction in the number of uterine glands and a decrement in the expression of morphoregulatory genes related to the Wnt signaling pathway, such as leukemia inhibitory factor (LIF) [49]. Likewise, a reduction of LGR4 levels results in downregulation of progesterone signaling in the uterus and affects the receptive state of endometrial luminal epithelial cells [50]. LGR4-deficient mice also show abnormal development of the Wolffian ducts and somatic cells with a decrease of WNT target genes lymphoid enhancer-binding factor 1 (LEF1) and AXIN2 [51]. An additional report shows that LGR4-deficient mice have a reduction in the corpus luteum enzymes for steroidogenesis as well as a decrease of common luteal marker genes associated with WNT-mediated EGFR-ERK signaling [52].

### 5.3. Eye

Different studies in mice have shown that LGR4 deficiency results in an eye-open phenotype at birth caused by problems in ocular embryonic development. This phenotype is associated with a reduction in EGFR activation and a decrease in proliferation and migration of keratinocytes from the eyelid epidermis [53,54,55]. Loss of LGR4 also leads to abnormalities in the development of the anterior ocular segment, including microphthalmia, iris hypoplasia, iridocorneal angle malformation, corneal dysgenesis, and cataract produced by a diminution of Pitx2, a direct target of the Gpr48-mediated cAMP-CREB signaling pathway [56]. Recently, it has been shown that LGR4 expression is regulated by the microRNA mir34a. Mir34a/LGR4 activity regulates cell proliferation, migration, and attachment of the ARPE-19 retinal pigmented epithelium cell line [34].

LGR4 has an important role in the induction of ocular cataracts. LGR4-deficient mice are prone to develop cataracts, showing early onset of lens opacification and a higher incidence of cataract formation. Mice with LGR4 deficiency had an increased sensitivity to environmental oxidative damage associated with a decreased expression of antioxidant enzymes such as catalase (CAT) and superoxide dismutase-1 (SOD1) which affect the redox state of the lens and contribute to cataract formation [57]. Furthermore, LGR4 also participates in the induction of cataracts mediated by let7-b. Dong et al. showed that let-7b targets LGR4 to regulate lens epithelial cell apoptosis, thus, facilitating cataract development [36].

### 5.4. Intestine

LGR4 is also an important mediator of intestinal development. LGR4-deficient mice show alterations in postnatal crypt development associated with defects in epithelial cell proliferation, Paneth cell differentiation, and downregulation of stem cell markers and Wnt target genes [24,58,59]. Likewise, knockout of LGR4 depletes the intestinal stem cells (Lgr5+/Olfm4+) and impairs proliferation and differentiation of the gut epithelium, associated with Wnt signaling [60]. As expected by these results, LGR4 is also essential to generate spheroids enriched in progenitor cells from the mouse fetal intestinal epithelium [61].

Another study demonstrated that LGR4 is involved in the maintenance of intestinal homeostasis. LGR4-deficient mice exhibited higher susceptibility to inflammatory bowel disease induced by DSS (dextran sodium sulfate) and higher mortality due to impaired proliferation and differentiation of intestinal crypts and Paneth cells during tissue regeneration. In addition, LGR4-null mice also showed decreased Wnt signaling, suggesting that LGR4/Wnt signaling acts as a protective factor against inflammatory bowel disease [62].

### 5.5. Mammary Gland

Recently, LGR4 has been implicated in mammary gland development and mammary stem cell regulation. Oyama et al. 2011 and Wang et al. 2013 demonstrated that LGR4-KO mice showed impaired mammary development. Loss of LGR4 resulted in impaired mammary morphogenesis, differentiation, and stem cell function. Mice lacking LGR4 exhibit defects of ductal elongation, branching morphogenesis, and reduction of terminal end buds as a result of disruption of mammary progenitor differentiation, low cap cell proliferation, and high apoptosis. Furthermore, LGR4-KO impairs stem cell self-renewal by inhibiting the Sox2 transcription factor through regulation of the WNT pathway [63,64].

### 5.6. Kidney

Several reports have shown that LGR4 is essential during renal development. LGR4-null mice showed low proliferation of kidney cells during development [60]. Kato et al. 2006 demonstrated that mice with LGR4 deficiency have renal hypoplasia, elevated concentration of plasma creatinine, and a decreased number and density of glomeruli [65]. An additional report showed that LGR4-KO mice have dilated renal tubules, impaired branching morphogenesis, and premature differentiation of the kidney, associated with GATA3 and LEF1 downregulation [66]. LGR4 deletion also resulted in increased apoptotic cells in the peripheral mesenchymal zone throughout kidney development [67]. Finally, LGR4-null mice have polycystic lesions in the kidney and fibrosis associated with an increment of polycystic kidney disease 1 (PKD1) and PKD2 gene expression, as well as abnormal expression of extracellular matrix proteins and activation of Wnt signaling [68].

### 5.7. Liver

LGR4 has an important role in liver function. In mice, LGR4 is expressed in hepatocytes that are responsive to RSPOs. RSPOs-LGR4 signaling reduces the expression of TNF-α, p65, and caspase-3, resulting in the reduction of cell death. In the same way, LGR4 knockout makes hepatocytes more vulnerable to TNF-α-induced cell death [69,70]. Furthermore, RSPOs-LGR4 signaling is an important mediator of metabolic liver zonation by enhancing the activity of a Wnt/β-catenin signaling gradient through all the liver. Loss of LGR4 decreases Wnt zonation and reduces hepatocyte proliferation also affecting the liver size. This suggests that LGR4 is an important liver regulator during development, homeostasis, and regeneration [71].

### 5.8. Epidermis

LGR4 also has an important role in the development of epidermal structures. LGR4 is expressed in the developing hair follicles and basal cells. Mohri et al. 2008, Kinzel et al. 2014, and Zak et al. 2016 showed that LGR4-KO mice exhibit impaired hair placode formation [60,72,73]. Mice without LGR4 have sparse head hair and focal alopecia behind their ears. This is associated with the reduction of EDAR, LEF1, and Shh expression, which are essential genes for hair follicle morphogenesis [72]. In addition, loss of LGR4 resulted in reduced epidermal thickness, low hair follicle numbers, and decreased proliferation of epidermal stem cells, indicating that LGR4 regulates hair follicle development [60]. Furthermore, Wang, 2010, reported that LGR4 induces keratinocyte cell proliferation and migration, associated with the induction of the HB-EGF ligand and EGFR/ERK/STAT3 signaling activation [74].

### 5.9. Teeth

LGR4 also participates throughout teeth development and regulation. LGR4 is highly expressed in the early stages of molar and incisor development in mice. Mice lacking LGR4 showed low SOX2+ dental epithelial cells, which can form molars, and LGR4 deficiency resulted in the absence of the third molar [75]. LGR4 is expressed in odontoblasts and Hertwig’s epithelial root sheath in mouse root formation. LGR4, through Wnt/β-catenin signaling, regulates proliferation and osteogenic differentiation of stem cells from apical papillae [76].

### 5.10. Bone

LGR4 has been implicated in bone development and remodeling. Luo et al. 2009 showed that mice without LGR4 had an important delay in osteoblast differentiation and mineralization of bone during embryo development. In the same way, loss of LGR4 affected postnatal bone remodeling, increasing the number and activity of osteoclasts through the cAMP-KPA-CREB-ATF4 signaling pathway [77]. Another study used the MCT3-E1 cell line to show that RSPO2-LGR4 signaling not only induced osteogenesis but also inhibited osteoclastogenesis through the Wnt signaling pathway [78]. In osteoblastic cells, LGR4 expression is induced by the BMP protein during osteoblast differentiation and activation [79,80]. Opposing to this induction, Cong et al. 2017 showed that microRNA-34c can inhibit LGR4 transcription, thus inducing osteoclast differentiation, regulating NF-κB and GSK3β signaling during that process [32]. Luo et al. demonstrated that LGR4 can interact with RANKL to negatively regulate osteoclast differentiation. They showed that LGR4 can compete with RANK for RANKL interaction, inhibiting RANKL/RANK canonical signaling pathway. Furthermore, when RANKL interacts with LGR4 it activates Gαq-mediated calcium signaling and suppresses NFATC1 during osteoclastogenesis [9]. Taken together, these studies provide evidence of the relevance of LGR4 in bone regulation, as LGR4 signaling can promote osteoblast activity and differentiation and inhibit osteoclast differentiation.

LGR4 also acts as a regulator of mesenchymal stem cells in the bone. Sun et al. 2018 found that LGR4 deficiency in mice decreased bone and fat mass by suppression of osteogenic and adipogenic differentiation of bone marrow stem cells. Additionally, they found that LGR4 increases proliferation while decreases migration and apoptosis of bone marrow stem cells. However, loss of LGR4 suppresses osteoblast differentiation and inhibits fracture healing [81]. Shi et al. 2017 showed that RSPO1 promotes osteogenesis of bone marrow mesenchymal stem cells through LGR4/Wnt/β-catenin signaling under mechanical stimuli [82]. Zhang et al. showed that LGR4 and RSPO3 regulate osteogenic differentiation of human adipose-derived stem cells. They showed that inhibition of LGR4 decreases osteogenic differentiation of adipose-derived stem cells and disrupts bone formation through inhibition of the ERK1/2 signaling pathway [83]. These studies indicate that LGR4 can regulate osteogenic differentiation of mesenchymal and adipose stem cells.

### 5.11. Additional Functions

LGR4 signaling has been identified in many additional normal and diseased tissues, and it is also associated with various cellular and physiological functions. Most studies focus on the role of LGR4 in developmental or adult pathological phenomena. For instance, LGR4 seems to be indispensable for efficient endoderm induction, gallbladder and cystic duct formation, and heart development, among other organs [84,85,86]. Moreover, mutations of LGR4 lead to developmental defects, such as aniridia-genitourinary anomalies-mental retardation syndrome [87], and they have been correlated with low bone mineral density and osteoporotic fractures [88]. It has even been shown that LGR4 deficiency results in delayed puberty [89].

Some other processes for which LGR4 is relevant are coronary artery development, myogenesis, and erythropoiesis, which are mediated by RSPOs signaling [90,91,92]. It has also been shown that LGR4, rather than LGR5, is indispensable in mammalian early hematopoiesis by downstream TGF-beta signaling [93]. In addition, LGR4 may play a critical role in wound healing, fibrosis, and inflammation [94,95], particularly through the NF-κB signaling [33,96]. LGR4 also takes part in the innate immune response by modulating the TLR2/4 pathway and regulating monocyte to macrophage differentiation [97,98].

LGR4 is also involved in central nervous system processes such as motor coordination and regulation of feeding behavior [99,100,101]. It has also been described that LGR4 plays an important role in the circadian regulation of plasma lipid levels. Moreover, LGR4 might be an adaptive regulator between lipid and glucose metabolism [102], and RSPOs-LGR4 signaling regulates cholesterol synthesis in hepatocytes [103]. In accordance with this, its activity is correlated with abdominal visceral fat accumulation [104,105]. In addition, LGR4 participates in blood pressure homeostasis as it improves aldosterone responsiveness and its expression levels are related to blood pressure control [106,107].

In summary, it has been demonstrated that LGR4 activity affects a plethora of cellular and physiological functions in several tissues. Further research might provide insights into the specific mechanistic processes that drive downstream LGR4 signaling and may offer opportunities for therapeutic intervention in various pathological conditions.

## 6. LGR4 in Cancer

Cancer is one of the leading causes of death worldwide. Cancer development involves genetic and epigenetic alterations that allow cells to escape from the mechanisms that control proliferation and survival. Many of these alterations correspond to signaling pathways that control multiple processes such as cell growth, cell death, cell fate, and motility [1]. Accumulating evidence indicates that LGR4 is upregulated in cancer tissues and is associated with the initiation, progression, and metastasis of a variety of cancers.

### 6.1. Breast Cancer

Breast cancer is one of the most common malignancies worldwide. This disease is the most commonly diagnosed neoplasm and is the leading cause of cancer-related deaths among females around the world [108]. LGR4 is over-expressed in breast cancer and it has been associated with poor prognosis. Patients carrying breast tumors with high expression levels of LGR4 have poor overall survival, decreased post-progression survival, reduced distant metastasis-free survival, and decreased relapse-free survival [109]. Loss of LGR4 results in decreased tumorigenic capacity, reduced cell proliferation, decreased migration, and impaired invasion and metastasis of breast cancer tumors [109,110]. Interestingly, Yuo et al. showed that LGR4 downregulation decreases the self-renewal potential of breast cancer stem cells, by regulating SOX2 expression and disrupting the EMT process through the modulation of the Wnt/β-catenin signaling cascade. In addition, LGR4 can modulate the FAK-SRC pathway and it regulates the actin dynamics and cell adhesion of breast cancer cells, thus promoting cell migration [109].

### 6.2. Colorectal Cancer

Colorectal cancer is the fourth most common cancer and the third leading cause of cancer-related deaths among both sexes [108]. Accumulating evidence indicates that LGR4 is highly expressed in colorectal tumors, especially in advanced tumors, metastasis, and metastatic lymph nodes [10,111,112]. Remarkably, LGR4 levels correlate with tumor stage and lymph node status, and high expression levels of this molecule are a poor prognosis factor for 5-year overall survival [112]. Moreover, the overexpression of LGR4 has been associated with higher invasion and lung metastatic capacity [111].

Gao et al. showed that LGR4 expression levels are inversely correlated with the levels of p27, a protein that acts as a negative regulator of the E2F transcription factor. They showed that the transcriptional activity of LGR4 is mediated by E2F in colorectal cancer [111]. In addition, LGR4 can mediate the signaling of β-catenin/TCF via regulation of GSK-3β phosphorylation through the MAPK/ERK1/2 and PI3K/Akt pathways in colorectal cancer [112]. A recent study showed that the circLGR4-derived peptide activates LGR4 to enhance WNT/β-catenin in colorectal cancer. Additionally, LGR4 is expressed preferentially in the cancer stem cell subset compared to non-cancer stem cells. CircLGR4, through LGR4, increased colon cancer stem cell self-renewal and enhanced their invasive and metastatic capacity [10]. Taken together, these reports suggest that LGR4 acts as a promoter of invasion and metastasis in colorectal cancer, and it modulates colon cancer stem cells by the activation of the WNT/β-catenin signaling pathway.

### 6.3. Lung Cancer

Lung cancer is the third most commonly occurring cancer and the first leading cause of cancer-related deaths among both men and women worldwide [108]. LGR4 is abundantly expressed in lung cancer adenocarcinomas [113,114] and tumors co-expressing both LGR4 and the RSPO3 ligand exhibit high aggressiveness. Interestingly, RSPO3 high expression levels have been associated with poor survival. RSPO3/LGR4 signaling enhances cell migration and invasion and promotes EMT by modulating the function of IQGAP1, a scaffold protein that binds LGR4 and leads the formation of the Wnt signalosome supercomplex [114]. Additionally, Yang et al. showed that LGR4 is targeted by mir-449b, which is downregulated in non-small cell lung carcinomas compared with normal tissues. Overexpression of mir-449b reduced proliferation and the invasive capacity of lung cancer cell lines by decreasing LGR4 expression [39], thus, highlighting the role of LGR4 in migration and invasion processes.

### 6.4. Oral Cancer

Oral cancer is a broad group of diseases occurring in any oral tissue. Oral cancer represents the seventeenth most common cancer among both sexes all over the world and fourteenth cancer in terms of mortality [108]. LGR4 exerts an influence on the progression of tongue squamous cell carcinoma [115,116]. It has been shown that both LGR4 and its ligand RSPO2 are over-expressed in tongue carcinoma. Notably, the high expression of RSPO2 is positively associated with advanced clinical stages, tumor size, and metastasis. High levels of RSPO2 decrease disease-free survival and increase the recurrence of patients harboring tongue squamous cell carcinomas.

LGR4 activation by RSPO2 binding enhances proliferation, tumorigenesis, invasion, and migration and it also increases EMT and stemness by activating the Wnt/β-catenin signaling pathway. Interestingly, the interaction of RSPO2-LGR4 increases the phosphorylation of LRP6 and DVL3, while it decreases the GSK-3β phosphorylation, leading to β-catenin translocation to the nucleus, thus inducing the expression of CyclinD1, c-Myc, and CD44.

### 6.5. Prostate Cancer

Prostate cancer is the second most frequently diagnosed cancer among men worldwide [108]. LGR4 plays an important role in prostate cancer progression. Luo et al. showed for the first time that high expression of LGR4 is associated with a shorter time of recurrence in patients with prostate cancer [117]. LGR4 inhibition decreases proliferation, invasion, migration, EMT processes, metastasis, and increases apoptosis of prostate cancer cells. Likewise, LGR4 over-expression increases tumorigenesis and decreases apoptosis. Interestingly, LGR4 inhibition affects tumorigenic capacity and metastasis in vivo in a prostate cancer murine model [37,110,117,118,119].

Liang et al., in 2015, showed that over-expression of LGR4 is associated with the up-regulation of Akt, a key effector of the PI3K/AKT signaling pathway, promoting tumor growth [118]. Recent evidence has shown that LGR4 over-expression increased the expression level of the androgen receptor, a transcription factor that controls PSA transcription and plays essential roles in prostate cancer progression. Interestingly, LGR4 facilitated the interaction of Jmjd2a, a histone demethylase of dimethylated lysine 9 H3, with the androgen receptor (AR) to increase PSA transcription [119].

A recent study showed that radiation treatment enhances the expression of both LGR4 and its ligands in AR-positive and negative prostate cancer cells. Remarkably, LGR4 inhibition confers radiation sensitivity only in AR-positive prostate cancer by regulating the activation of CREB1, a transcription factor that promotes DNA repair [120].

It is interesting to note that LGR4 expression can also be targeted by some microRNAs in prostate cancer cells. Li et al. showed that the expression of miR228 disrupts IL6-mediated prostate cancer tumorigenesis via suppression of LGR4 expression [37]. MiR-137 also directly targets LGR4 and inhibits migration, invasion, and EMT through the EGFR/ERK signaling pathway [38].

### 6.6. Skin Cancer

LGR4 plays crucial roles in skin carcinogenesis and melanoma development. LGR4 is over-expressed in melanoma cells; however, it is barely expressed in squamous and basal cell carcinomas [121,122]. LGR4-deficient mice show retarded skin tumors and smaller tumor structures compared to wild-type mice. LGR4 deficiency reduces hyperplasia and keratinocyte proliferation by decreasing the Wnt/β-catenin and MEK/ERK signaling pathway [122]. Strikingly, LGR4 activity has also been shown to enhance keratinocyte proliferation via EGFR/ERK/STAT3 signaling [74]. Recent evidence has shown that LGR4 expression is regulated by mir-34a. Overexpression of mir-34a attenuates migration, invasion, and EMT by decreasing LGR4 expression, which regulates the expression of the matrix metalloproteinase 2 (MMP2) in melanoma cell lines [121].

### 6.7. Other Cancers

Overexpression of LGR4 has also been observed in glioblastoma, osteosarcoma, gastric, ovarian, and thyroid carcinomas [123,124,125,126,127,128]. Interestingly, high levels of LGR4 can promote the proliferation of glioma and gastric cancer cells, probably due to Wnt/β-catenin activity [110,123]. Moreover, high levels of LGR4 have been associated with poor overall survival and recurrence-free survival in ovarian cancer [125]. In thyroid cancer, upregulation of the LGR4/RSPO2 pathway leads to tumor aggressiveness, promoting cell proliferation and migration through the Wnt/β-catenin pathway and MAPK/ERK1/2 signaling [124]. In osteosarcoma, Liu et al. showed that STAT3 binds to the LGR4 promoter region in response to IL-6 and promotes its transcription [126].

A recent study associated the role of LGR4 with the regulation of immune cells in the tumoral microenvironment. LGR4 can promote tumor-associated macrophages (TAMs) M2 polarization due to the activity of RSPOs/LGR4/ERK/STAT3 signaling. TAMs are the largest leukocyte population found in the tumoral microenvironment. These cells have high plasticity and can polarize to M1-macrophages with pro-immunological activity, or M2-macrophages with immunosuppressive activity and a role in tumor immune evasion. Researchers showed that RSPOs/LGR4-inhibition with a soluble LGR4 extracellular domain (LGR4-ECD) or an RSPOs neutralizing antibody attenuates M2-TAMs polarization and it enhances the anti-tumor activity of CD8^+^ T-cells [129].

## 7. LGR4 Function in Cancer Stem Cells

It is well established that tumors harbor a functional subpopulation of cells that exhibit similar characteristics to normal stem cells. These cells, called cancer stem cells (CSCs), can indefinitely self-renew and differentiate into phenotypically diverse cells [130]. CSCs are thought to initiate and sustain tumor growth, enhance metastasis, and provoke tumor relapse and chemoresistance in many types of cancer [131]. The properties of CSCs are regulated by specific cell signaling pathways that control the self-renewal and differentiation of stem cells.

LGR4 is a key molecule that can regulate both normal and cancer stem cells. Recently, studies have demonstrated that LGR4 enhances the properties of CSCs in many types of tumors. Yue et al. showed for the first time that LGR4 regulates the breast CSC population. Loss of LGR4 reduced tumorigenesis and lung metastasis by affecting the CSC population in MMTV-PyTM mice. Depletion of LGR4 resulted in a reduction of SOX2+ breast cancer stem cells, disruption of the Wnt/β-catenin signaling pathway, and a decrease of the tumorsphere formation ability [109]. Zhang et al. showed that RSPO2/LGR4 signaling can modulate the properties of tongue CSCs. Tongue squamous cell carcinoma cell lines treated with RSPO2 have higher numbers of the CD44+, CD133+, and ALDH+ CSC population, possess a high expression of CSC markers (CD133, OCT4, SOX2, and CD44), and exhibit increased sphere formation ability [115]. A recent study showed that LGR4 regulates the epithelial cancer stem cell subpopulation in ovarian cancer. Knockdown of LGR4 resulted in the inhibition of stem cell transcription factors (POU5F1 and SOX2), reduction of cell surface markers (CD133, ALDH1A2), and suppression of tumor growth and metastasis of ovarian cancer cell lines. Interestingly, LGR4 maintained cancer stem cell features and promoted tumor growth and metastasis through ELF3, an epithelium-specific transcription factor. Mechanistically, LGR4 and ELF3 form a reciprocal regulatory loop positively modulated by WNT7B/FZD5 via the non-canonical Wnt signaling pathway, thus maintaining the stemness of ovarian cancer cell lines [128].

Recent evidence showed that LGR4 regulates colon cancer stem cells by interacting with the circLGR4-peptide ligand and inducing the Wnt signaling pathway. CircLGR4 is preferentially expressed in advanced colon tumors and in CSCs, where it encodes a peptide with essential functions for the CSC population. Inhibition of either circLGR4-peptide or LGR4 decreased the proportion of CSC population, and impaired sphere formation capacity, invasion, and migration. Over-expression of circ-LGR4 enhances the properties of CSCs, however, circ-LGR4 mutants failed to regulate CSCs, thus confirming its critical role in colorectal CSCs [10].

LGR4 is also essential for leukemic stem cell self-renewal in acute myeloid leukemia (AML) patients. RSPO3/Lgr4 upregulates WNT/self-renewal target genes to block differentiation, which contributes to the aggressive leukemia phenotype, probably through the pCREB-CBP complex. Interestingly, disrupting the RSPO3-LGR4 interaction using an anti-RSPO3 antibody (rosmantuzumab) reduced the leukemia burden by promoting differentiation and impairing the self-renewal of stem cells in AML patient-derived xenografts without affecting normal hematopoietic stem cells. This study highlights the therapeutic value of targeting LGR4 signaling to impair stemness in AML [132].

Accumulating evidence has revealed that LGR4 can expand the fraction of CSCs due to its interaction with RSPOs ligands or with the circLGR4-peptide, thus enhancing the Wnt/β-catenin signaling pathway. However, the function of LGR4 mediated through other ligands such as RANKL is still unexplored. LGR4 might act in a Wnt/β-catenin independent way exerting different functions in the regulation of CSCs; however, more studies are needed to completely understand the complex functions of LGR4.

## 8. Conclusions

LGR4 signaling regulates a plethora of cellular processes by interacting with various ligands such as RSPOs, Norrin, circLGR4-peptide, and RANKL. The importance of LGR4 and its ligands in the development, physiology, and maintenance of many organs has been well established in a variety of in vivo and in vitro studies. In mice, Lgr4 knockout is associated with embryonic lethality and surviving animals display severe developmental deficiencies in multiple organs and tissues. LGR4 is also involved in the regulation of cell proliferation, survival, and differentiation of several tissues.

LGR4 has also been associated with cancer progression, cell migration, and invasion, mainly through the activation of the Wnt/β-catenin signaling pathway. Several studies support the idea that inhibitors of the RSPOs/LGR4/Wnt/β-catenin axis could reduce tumor progression, metastasis, and recurrence. There is also strong evidence that LGR4 is a key regulator of the cancer stem cell population in different types of cancer. LGR4 expands the proportion of CSCs, enhances self-renewal, and increases the sphere-forming ability of CSCs. Interestingly, disruption of the LGR4 signaling pathway results in a decreased population of CSCs, reducing tumorigenesis, affecting sphere-forming ability, and impairing the migration and invasion of CSCs.

Although significant progress has been made in the molecular comprehension of how LGR4 interacts with RSPOs to potentiate the Wnt/β-catenin signaling pathway, many questions remain elusive. The role of LGR4 signaling through its newly discovered ligand (RANKL) in the homeostasis of most tissues and their implication in cancer is still unknown. Recent evidence suggests that the LGR4/RANKL signaling activates independent pathways that could impact tumor development and stem cell behavior. The understanding of the role of the RANKL/LGR4 signal is essential for exploring the complex roles of LGR4 in physiological and pathological processes.

## Abbreviations

ALDH	Aldehyde dehydrogenase
AML	Acute myeloid leukemia
APC	Adenomatous polyposis coli
ATF4	Activating transcription factor 4
BMP	Bone morphogenetic protein
cAMP	Cyclic adenosine monophosphate
CAT	Catalase
CK1	Casein kinase 1
CREB	cAMP response element-binding protein
CSCs	Cancer stem cells
DSS	Dextran sodium sulfate
DVL	Dishevelled
EDAR	Ectodysplasin A receptor
EGFR	Epidermal growth factor receptor
ELF3	E74 like ETS transcription factor 3
EMT	Epithelial-mesenchymal transition
ERK	Extracellular signal regulated kinase
ESR1	Estrogen receptor 1
FAK	Focal adhesion kinase
FSH	Follicle-stimulating hormone
FZD	Frizzled
GPCR	G protein-coupled receptor
GSK3β	Glycogen synthase kinase 3 beta
HB-EGF	Heparin-binding EGF-like growth factor
IL-6	Interleukin 6
INSL3	Insulin-like peptide 3
IQGAP1	IQ motif containing GTPase activating protein 1
JMJD2A	Jumonji domain-containing protein 2A
LEF1	Lymphoid enhancer binding factor 1
LGRs	Leucine-rich repeats containing G protein-coupled receptors
LH	Luteinizing hormone
LIF	Lymphoid enhancer-binding factor 1
LRP	Low-density lipoprotein receptor-related protein
LRR	Leucin-rich repeats
MAPK	Mitogen-activated protein kinase
MMP2	Matrix metalloproteinase 2
NFATC1	Nuclear factor of activated T-cells, cytoplasmic 1
NF-κB	Nuclear factor of activated T-cells, cytoplasmic 1
OLFM4	Olfactomedin 4
OPG	Osteoprotegerin
PDK	Polycistic kidney disease
PI3K	Phosphatidylinositol 3-kinase
PITX2	Paired Like homeodomain 2
PR	Progesterone receptor
PSA	Prostate-specific antigen
RANK	Receptor activator of NF-kappa B
RANKL	Receptor activator of NF-kappa B ligant
RNF43	Ring finger protein 43
RSPOs	R-spondins
RXFP	Relaxin family pepetide receptor
SHH	Sonic hedgehog
SOD1	Superoxide dismutase-1
STAT3	Signal transucer and activator of transcription 3
TAMs	Tumor-associated macrophages
TCF	T-cell factor
TGF-β	Transforming growth factor beta
TLR	Toll-like receptor
TNF	Tumor necrosis factor
TSH	Thyroid-stimulating hormone
TSR	Thrombospondin 1 repeat domain
ZNRF3	Zinc and ring finger
